# The challenges and gains of delivering a home-exercise intervention: a qualitative study of physiotherapists and physiotherapy assistants

**DOI:** 10.1186/s12891-022-05790-z

**Published:** 2022-09-03

**Authors:** Karen L. Barker, Jon Room, Francine Toye

**Affiliations:** 1 Physiotherapy Research Unit, Nuffield Orthopaedic Centre, Oxford University Hospitals NHS Foundation Trust, Windmill Road, Oxford, OX3 7HE UK; 2 Nuffield Department of Orthopaedics, Rheumatology and Musculoskeletal Sciences, University of Oxford, Oxford, UK

**Keywords:** Qualitative research, Knee arthroplasty, Physiotherapy, Physiotherapy assistant

## Abstract

**Objectives:**

The paper presents insights from the Community based Rehabilitation after Knee Arthroplasty (CORKA) trial. We aimed to explore physiotherapists and physiotherapy assistants’ experiences of delivering a home-base exercise intervention following knee replacement surgery. We were particularly interested in the feasibility, potential benefits and barriers of a community-based exercise programme from the perspective of physiotherapists and physiotherapy assistants and to understand any constraints or training needs that arose.

**Design:**

Qualitative thematic analysis of semi-structured interviews.

**Setting:**

The Community based Rehabilitation after Knee Arthroplasty (CORKA) trial.

**Participants:**

Five physiotherapists and six physiotherapy assistants with a range of clinical experience.

**Methods:**

Interviews were digitally recorded and transcribed verbatim. We used the stages of reflexive thematic analysis suggested by Braun and Clarke. One researcher conducted the interviewers whilst three researchers with experience in qualitative research methods contributed to the coding and analysis of data.

**Results:**

We developed seven themes that help to understand the benefits and challenges of delivering treatment interventions in a person’s home: seeing the person in their own world; thinking outside the cubicle;developing people skills; enjoying the above and beyond; treading a fine line between patient and friend; feeling outside my comfort zone; needing a support network.

**Conclusions:**

Treating people in their own homes facilitates a holistic approach. Our findings highlight areas for clinical education: (1) how do we help clinicians to tread the fine line between friend and professional (2) how do we balance the need to provide support and structure with the freedom to work creatively and independently?

**Supplementary Information:**

The online version contains supplementary material available at 10.1186/s12891-022-05790-z.

## Introduction

Over 100,000 primary knee replacements are undertaken each year in the United Kingdom (UK) [1]. Although most patients achieve a satisfactory outcome, many patients continue to report poor outcomes after knee arthroplasty. Given the rising number of these operations, the relatively limited therapy resources available, and the increasing age and frailty of patients receiving joint arthroplasty, it is important to concentrate rehabilitation resources on those patients who need the most help to achieve a good outcome. Post-operative physiotherapy is an important part of recovery which may be delivered in a traditional out-patient clinic setting or in the patient’s own home. The Community based Rehabilitation after Knee Arthroplasty (CORKA) trial was a prospective, individually randomised two-arm controlled trial with blinded outcome assessment for the clinical outcomes at baseline, 6 and 12 (primary outcome) months. It aimed to determine whether a home-based multi-component rehabilitation programme provided to patients deemed at risk of a poor outcome following knee arthroplasty using a bespoke screening tool was better than usual out-patient based care. The CORKA trial rehabilitation package was delivered by physiotherapy assistants with supervision from qualified physiotherapists [2, 3].

In the UK, The National Health Service (NHS) Long Term Plan has identified the need to decentralise services, with greater community provision and less emphasis on care provided in large acute hospitals unless patient clinical need makes hospital based care clinically mandated [4]. We suggest that the home-based intervention used in the CORKA trial is a model that meets this strategy as it targets care to those at higher risk who most need it, within participants’ own homes and communities. It addresses the workforce shortage issue by using an innovative workforce model of advanced rehabilitation assistants, moving UK service provision closer to that which has been proven to be effective in North America, where the use of physical therapy assistant graded staff is well embedded and where trials comparing the delivery of exercise programmes by qualified physical therapists or physical therapy assistants supervised by qualified physical therapists have demonstrated equal outcomes [5]. The use of different workforce models and particularly a hybrid model of assistants supervised at a distance by qualified therapists has not been researched in any depth in the UK. However, it is an emerging service delivery model in the UK and elsewhere, where the need to provide greater levels of care to meet the needs of an ageing population is set against a backdrop of insufficient commissioned training places for students [6].

Published guidance from professional bodies on competency training and roles that may be allocated to assistant staff can support advanced or experienced physiotherapy / rehabilitation assistants to deliver effective protocol-driven care [7]. This will allow prioritisation of qualified staff time for more complex care and management of the overall patient pathway. This model is more advanced in speech and language therapy where randomised controlled trials have compared the roles of speech therapy assistants and qualified therapists undertaking speech therapy for children in schools and for swallowing practice [8, 9].

In this qualitative study we aimed to understand the gains and challenges of delivering a home-based exercise intervention from the perspective of physiotherapists and physiotherapy assistants to assess the feasibility and any barriers to delivering care in the community setting with this workforce model [2, 3].

## Methods

We used the Equator standards for reporting qualitative research (SRQR) to enhance the transparency of our report [10] (Additional file 1: Supplementary file). Potential participants were identified by the CORKA trial coordinator and given an information sheet about the study. Physiotherapists and rehabilitation assistants who were involved in delivering the CORKA arm of the trial intervention were contacted by participant invitation letter and invited to participate. Those who responded that they were interested in taking part were contacted by the qualitative researcher to arrange a convenient interview. The same researcher completed all interviews; FT is an experienced anthropologist who is a qualified physiotherapist. She was supported in the analysis by two qualified physiotherapists experienced in qualitative research and trained in interview methods [JR, KB].

An interview guide was used flexibly with follow up prompts to ensure that relevant areas were covered and that participants could introduce new relevant areas (Additional file 2). Two interviews with rehabilitation assistants took place on the telephone, and the rest of the interviews took place in a quiet room at work. We conducted the research using a reflexive thematic analysis approach [11]. Interviews were digitally audio recorded and transcribed. Recruitment was stopped when a wide range of views had been obtained and no new topic areas were being raised. We transcribed the interviews and completed initial coding iteratively and met as a team regularly to review emerging themes and to discuss areas that might be further explored. During these meetings we made the joint decision to stop data collection when we felt that we were no longer uncovering new perspectives or themes. We uploaded the full interview transcripts to Nvivo software designed to assist qualitative analysis.

We followed the six phases of analysis outlined by Braun and Clarke [12], first familiarising ourselves with the data by reading and re-reading the transcripts (stage 1), followed by generating codes to capture key codes or labels (stage 2).The first three transcripts were independently coded and discussed by two researchers. As similar coding units were identified, a single researcher coded subsequent transcripts. A single researcher constructed potential themes (stage 3) by comparing the initial codes and collating them to develop broader patterns of meaning. These potential themes, along with their codes and data were reviewed and refined by two other researchers (phase 4) to define and name the final themes (stage 5), prior to writing up and developing an overarching conceptual model (stage 6). The aim of this process was to discuss, and decide upon, a description of each theme and confirm that the themes were supported by the data. The process of constantly comparing data, codes and themes occurred throughout the analyses and was recursive rather than strictly sequential with the team meeting regularly to discuss. Rigour was therefore promoted through collaboration [13].

## Results

We interviewed five physiotherapists and six physiotherapy assistants. The interviews lasted between 1–1.5 h and were audio recorded and transcribed verbatim. The number of years worked post-qualification for physiotherapists ranged from 1–32 years, and assistants had worked in their role between 1-30 years. This allowed participants to draw on a wide range of experiences in a caring role. Full details of the demographics of the interviewees are shown in Table 1.

**Table 1:** Description of interviewees

Code	Designation	Gender	Age	Years Experience treating Knee arthroplasty
P1	Physiotherapist	Female	55	32
P2	Physiotherapist	Female	43	22
P3	Physiotherapist	Male	37	16
P4	Physiotherapist	Male	22	1
P5	Physiotherapist	Female	33	10
PTA1	Physiotherapy Assistant	Female	56	30
PTA2	Physiotherapy Assistant	Male	24	3
PTA3	Physiotherapy Assistant	Female	23	3
PTA4	Physiotherapy Assistant	Male	34	5
PTA5	Physiotherapy Assistant	Male	23	1
PTA6	Physiotherapy Assistant	Female	22	2

**Fig. 1:**
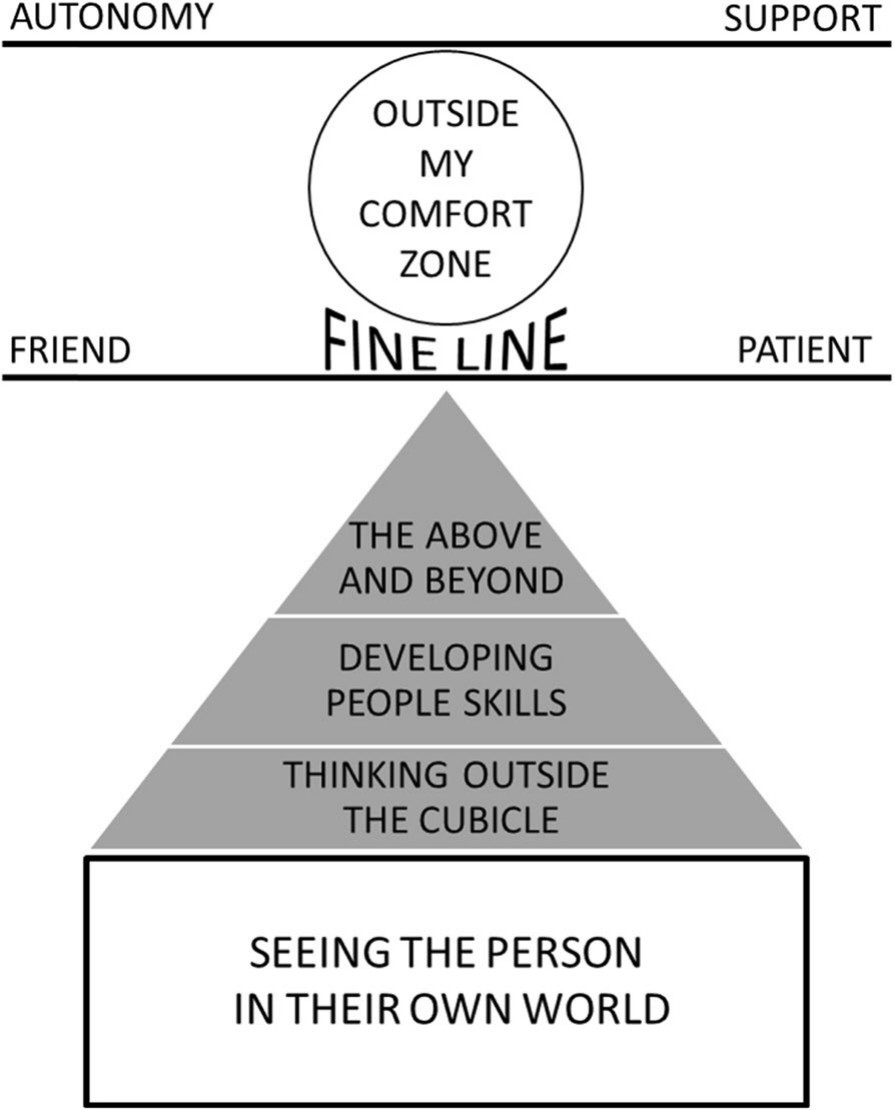
Visual representation of themes

We developed seven themes that help to understand the benefits and challenges of delivering treatment interventions in a person’s home: (1) seeing the person in their own world; (2) thinking outside the cubicle; (3) developing people skills; (4) enjoying the above and beyond; (5) treading a fine line between patient and friend; (6) feeling outside my comfort zone; (7) needing a support network. Our findings, summarised in Fig. 1, illustrate how home-based therapy was underpinned by a relational and holistic approach (seeing the person in their own world). This approach relied on individualised care (developing people skills) and creativity (thinking outside the cubicle); and could lead to both professional and personal gains (enjoying the above and beyond). Participants could find it challenging to manage the professional boundary (treading a fine line between patient and friend), and geographical distance from the medical setting could make them feel vulnerable (feeling outside my comfort zone). At these times, the right level of support from colleagues, which made them feel supported yet still autonomous, was important (balancing support and autonomy).

We illustrate these ideas below with narrative exemplars.

### Seeing the person in their own world

This theme describes the value of seeing the person in the context, and complexity, of their own world. Participants recognised the need for a ‘holistic’ encounter.

I think that holistic approach is really important: Yes, we’re there to get their knee bending . . . but in the bigger picture, I want them to be able to go outside and *use* their knee . . . to go and see friends, or do dancing . . . I saw quite a nervous chap . . . most of the time, we didn’t spend doing exercises . . . his knee was functionally really good . . . getting back to work was his main worry. So, we spent a lot of time talking about how to get back to work . . . the kind of holistic approach for him was very important (Physiotherapist)

Some compared the encounter in a medical setting, and its focus on the body part, with a more relational encounter at home. Entering a person’s home was described as a privilege, where the balance of power can shift from clinician to patient: the *patient* becomes *person*.

[being at home] sort of takes it out of that context where the mindset of you’re just there just to fix you . . . you’re with that chance to be able to talk with people, they’re able to see you as being human. (Assistant)

You’re in there and it’s a privilege to be in someone’s home. They’ve let you in. You’re not just seeing them; you’re seeing everything really . . . you probably will go to their bedroom to do some exercises on the bed, into their kitchen . . . So, it’s quite invasive into their world, but it also gives you a lot more in terms of the whole picture. (Physiotherapist 52)

Participants felt that in their future clinical practice, they would focus more on striving to ‘see the person’, suggesting a change in future clinical encounters.

Thinking about the patient as a whole - I think is what I’ll take forward . . . That’s been one of the key things that I’ve taken away. (Physiotherapist)

I would say that I’d strive now to see the person as an individual . . . . to try and just understand where that person’s coming from, because even with something that you think is as simple as a knee replacement . . . the impact on that one person’s life . . . You don’t just know that stuff instinctively, you’ve gotta know the person. (Physiotherapist)

### Developing people skills

This theme describes the benefits of ‘people skills’ which allow you to enter a person’s world and provide individualised, and therefore effective, care. Participants recognised the therapeutic potential of these skills.

To be truthful they need to have good people skills to be able to talk to people and not talk down to them . . . don’t make ‘em feel like they’re on detention . . . personal skills count a lot . . . You’ve sort of got to win their confidence and once you do that it’s amazing what you can get out of them . . . get that then you’re rockin’ and rollin’. (Assistant)

Good people skills meant getting to know the person in their context and having the flexibility to respond to individual needs.

The reality is that things change quickly, you have to be pragmatic . . . real life is wonderfully, beautifully messy.. . You can’t know a thing that’s gonna come up. (Physiotherapist)

It’s just making that patient feel really comfortable . . . letting them know that it’s okay and everyone doesn’t follow the same rehab path . . . I think you have to realise you have to be very flexible around the patient. (Assistant)

### Thinking outside the cubicle

This theme describes how seeing the person in a real world in situ can enhance creative thinking. Participants shifted from a ‘theoretical’ and clinical approach to a ‘real world’ situational approach.

in a sort of sterile clinic or environment where the floor’s totally flat, there’s no obstacles . . . it doesn’t bear that much resemblance to somebody’s house . . . I think seeing people in their own home, it’s just different . . . you can *see i*t, it’s not just theoretical. (Physiotherapist)

Participants enjoyed the freedom to be less prescriptive, to use their imagination, and to be creative ‘outside the cubicle’. This creative and less prescriptive approach was contrasted to the clinical space.

I enjoyed being able to give the people the realisation that you don’t need any fancy equipment . . . utilising the equipment that they’ve got, their chairs, their stairs . . . a bit of rope that the husband has got in the shed . . . It was taking it away from just ‘here’s a sheet with some exercises on it’ . . . the skills of being able to adapt to the situation. (Assistant)

[in the clinical space] you probably can’t build up as much of a picture, and you can’t see the home environment that you’re working with . . . we’re able to kind of walk through a day in the life, rather than just talk through it . . . thinking about the bigger picture . . . doing a bit of detective work. . .. I think [we] probably get a bit trapped in the cubicle thinking . . . fix the problem, next patient, fix the problem, next patient . . . (Physiotherapist)

### The *above and beyond*

This theme describes the personal gain from having a real impact on people’s lives. Participants recognised that both patient and therapists gained from a more holistic encounter, and this might reduce professional ‘burn-out’.

I gained . . . personally . . . building relationships; being able to gain more of an insight into human beings . . . this has been really important to me. To be able to spend the time talking to people and understanding what makes them tick . . . I want to take on the stories . . . I think if I wasn’t doing that . . . I’d be less satisfied and more likely to burn out . . . that’s the bit that I enjoy the, the above and beyond. (Physiotherapist)

There was a sense that the gains of this were mutual, benefitting patient and therapist, and some described a profound sense of camaraderie.

It was so lovely walking around that big park, seeing her in her community and she had a lump in her throat, ‘I never would have been able to do that three months ago, you have no idea what that surgery’s done for my life’. And you know she squeezed my hand and said, ‘that’s great thank you’. And she didn't have much honestly . . . she had nothing. It was great. So thank you. (Physiotherapist)

### Treading a fine line between patient and friend

This theme describes the challenge of managing the boundary between yourself and your patient. Participants described a tension between developing a good rapport and the need to maintain a professional boundary.

You always have to realise that the patient . . . is a patient and not a friend . . .? there is that fine line . . . [be] clear to them that you’re here to rehab them . . . some patients want to have a laugh and a joke with you, but I wouldn’t say that is unprofessional . . . it’s polite to have that five-minute conversation . . . and then once that’s out the way, get on with the treatment. (Assistant)

Building rapport is really important to get the patient engaged, but it’s managing the professional relationship is the difficult bit. . . You do get to you know their family, you meet their kids, you know their dogs’ names . . . and you obviously share your own life stories . . . I found it kind of difficult to negotiate that barrier sometimes . . . because they feel like you’re now friends . . . so it’s difficult to steer back from a close to a professional relationship. (Physiotherapist)

There was a sense that the professional ‘line’ was blurred and that you could easily cross the line and share too much. Participants describe a ‘middle area of closeness’ where you enter a person’s world far enough, but not so far that you become ineffective.

Doing a bit of a share . . . *appropriately* . . . it breaks down barriers very quickly, doesn’t it . . .? It is knowing where to stop . . . not sharing *too much* . . . I think it’s very blurred . . . sometimes you might cross that line. (Physiotherapist)

I think there is kind of a middle area of closeness . . . it’s not the more you get into their lives, the more help you can offer . . . there’s a threshold that helps, and then maybe as you get too far, you’re not objective . . . I think you can get a lot from [talking] but if you go to the extreme . . . you hardly have any impact in terms of your knowledge. (Physiotherapist)

Participants described situations where it might be more challenging to walk this fine line. For example, if you felt more of a connection with someone.

Some people you do sort of click with . . . I got to the point where I liked him, he was a nice bloke . . . I can understand where you’re coming from, you used to be fit and healthy . . . now you can hardly walk . . . It’s sort of seeing somebody sort of knocked back quite severely . . . . I felt that I want to be able to help him as much as I could. (Assistant)

### Feeling outside my comfort zone

This theme describes a feeling of vulnerability when working at a geographical distance from a medical setting.

They were sort of under the impression that I was a physio . . . ‘What do you mean, you’re not a physio?’ . . . and it just sort of made me feel a little bit, uncomfortable . . . there was a husband firing a lot of questions at me . . . it made me get my back up a little bit . . . I don’t really like it when he’s sort of interrogating me. (Assistant)

There was a sense that it was very important to have access to support from colleagues. For example: severe pain, swelling, lack of expected progress, or if things just ‘didn’t look right’.

It was just mainly with the people who weren’t progressing as much . . . the lady who wasn’t getting beyond 30 degrees of movement . . . . I felt I’d sort of done everything that I could. So, of course, I’d go back and touch base with [the physio] . . . even [the physio] was a bit baffled. (Assistant)

Physiotherapists recognised that this could be extremely challenging for assistants and emphasised the need for communication and support.

I think the important thing would be just to emphasise openness and communication . . . ‘Don’t sit on something: if you’re worried about something, tell me . . . I might be worried about it as well, and I’ll tell someone else’ (laughter) . . . report it . . . discuss it . . . have a conversation. . . reason it through . . . give your point of view . . . I guess it’s wanting to have trust. (Physiotherapist)

### Balancing support and autonomy

This theme describes the challenge of providing the right level of support whilst also encouraging creative and independent decision making.

It’s quite nice . . . to feel like you’re making an impact independently . . . But at the same time, it’s nice to have that support . . . I didn’t feel at all like I was kind of abandoned or deserted . . . I think if the physio was coming in every single time they’d be pressure . . . are they kind of judging [me]? (Assistant)

There was a sense that a collaborative and supportive partnership between physiotherapist and assistant provided a safe place to learn.

I took on a lot of skills and I learnt a lot about myself, I learnt a lot more about how to present myself to a patient because I was independent, and I was learning from the [physios] . . . so, I was pitching the best skills from everybody and putting them into what I want to become. (Assistant)

## Discussion

The purpose of this study was to explore the gains and challenges of delivering a home-base exercise intervention from the perspective of perspective of physiotherapists and assistants, focusing on the differences of working within patients’ own homes. The context of the patients’ own homes as the place where the therapeutic interventions were delivered presented an assemblage of medical, social, environmental and psychological features. It is standard practice within the UK for out-patient physiotherapy to be delivered in a clinic setting by registered qualified physiotherapists. In contrast, the CORKA home-based intervention was multidisciplinary in content, delivered in participants’ own homes, and used a staffing model of rehabilitation assistants supervised by a qualified physiotherapist.

We found that both physiotherapists and physiotherapy assistants were positive about working with each other in the community and that themes cut across both professional groups. Our findings indicate that treating people at home facilitates a holistic approach benefitting from ‘seeing the patient in their own home’. This was valued by the staff with a recognition that the purpose of rehabilitation is to help patients to function and live an active life participating in activities within their own environment; arguably making a home based intervention more appropriate than the clinic setting; with an improved prospect of achieving longer term social and community participation through confidence gained from exercise within the home setting [14].

Interviewees also reported that working within the patents’ homes made them ‘think outside the cubicle’ offering more innovative and functional based exercises as they used the context of the home to make exercises more tailored and using the unfamiliar context in the patients’ homes by changing their roles in response to the patient’s needs and desires. Thus, treatment goals could be more closely linked to the participation level of the International Classification of Impairments, Disabilities and Handicaps (ICDH) hierarchy [15]. This finding is in agreement with Van Koch et al. who observed and compared treatment settings between the home and clinic in stroke rehabilitation reporting a clear difference in roles adopted by therapists working within patients’ homes, with a more active role played in directing the treatment by the patient when treated in the home setting [16].

Our study also indicates that participants had to utilise different communication and soft skills requiring greater people skills, flexibility and emotional intelligence as the balance of power had shifted from them being ‘in control’ within the hospital setting to being a guest in the patient’s home and thus feeling a change in the balance of power. Physiotherapists and assistants spoke about how they had to establish rapport and trust in order to work with patients by invitation rather than by right. They also enjoyed the challenge of having to use greater flexibility to adapt to changing circumstances when faced with differing levels of progress, co-operation of emerging problems. They enjoyed the aspect of getting to know patients better but also reported it took a different skill set to treat by invitation rather than by right as in the structured hospital setting. This resonates with the findings of Heckman and Cott who reported that physiotherapists recognised the different client-physiotherapist relationship and embraced the shift in control rather than perceiving it as a threat [17]. They felt a degree of personal satisfaction from seeing that they were having an impact on the patients’ lives. Our findings indicate that that personal skills, and not simply professional knowledge, are integral to compassionate and effective clinical practice. The home therapy environment encouraged clinicians to develop these skills, and the clinicians indicated that they would transfer these skills to other clinical settings. Beyond the therapeutic gains, the clinicians described personal rewards of working ‘above and beyond’.

Our findings highlighted areas where staff found the treatment setting more challenging. These related to two key areas of professionalism firstly the need to behave differently when delivering care in a patient’s home compared to in the clinic environment utilising different skills, communication strategies and relying on greater soft skills, which are integral to effective care. They also described the challenge of occupying multiple roles being within the same session an educator teaching exercises; a professional assessing progress and when to progress or limit further challenge; but also, a friend when they were sharing a cup of tea or talking about family. Some interviewees, particularly those who were less experienced reported a tension between developing rapport and maintaining professional boundaries.

Finally, some participants explored the felt out of their comfort zone working away from the structure of the hospital and needed strong communication links with the team. However, this needed to be delivered in a manner that supported but did not challenge autonomy so that each person felt able to deliver the treatment programme in a collaborative manner, adapting as the situation in front to them developed, but also knowing that they have colleagues to discuss and problem solve with.

The transition from hospital to home-based care can be challenging and in nursing it has been recognised that the transition can be a culture shock for those more used to a highly specialised setting [18]. This raises the challenge for clinical educators of how to equip students with the skills needed to tread the fine line between being seen as a clinician / friend by the patient and being viewed as a professional. Secondly, how to balance the need to provide support and the freedom to work creatively and independently? Currently there is no standardised training in communication skills for either undergraduate physiotherapy programmes or for assistants. There is recognition that enhancing communication skills is important, in particular to stop communication being seen as an act done to the patient by the physiotherapist rather than a reciprocal collaborative activity [19]. There is a perception that these skills of communication and emotional intelligence are soft skills and peripheral, whereas qualitative work interviewing patients shows that they are key and underpin the therapeutic encounter, encouraging an integrative encounter and a more inclusive, satisfactory patient-clinician dynamic. Muddle et al. conducted a systematic review of the literature describing communication skills training in physiotherapy education but found that the effectiveness of this was difficult to establish due to the poor quality of the published work and lack of detail in the intervention reporting [20]. Our results indicate that both professional groups would have benefitted from learning and education in the skills of working within a patient’s own home and the differences in the professional relationship when you become a ‘visitor’ rather than a ‘host’ in a clinical setting where the power balance is different. We suggest that this is an important area for clinical education and whilst considerable research activity has been devoted to investigating tele-medicine and developing frameworks of core capabilities the same does not exist for face-to-face communication skills [21].

The key message of our study is that working within the patients’ homes required a broader skill set from both physiotherapists and assistants than traditional core knowledge and this required them to develop an array of professional craft knowledge. This tacit, skillset was crucial to establishing an effective therapeutic encounter. In both education and clinical practice there is a need to acknowledge the importance of professional craft knowledge to supplement existing skills and to explore how the recognition and transmission of these craft skills can be taught and facilitated [22]. In order to deliver optimal patient care educators and researchers need to navigate the validity of utilising evidence- based research derived practice alongside the acceptance of professional craft experiential learning. To date, no such framework exists to facilitate effective knowledge translation and performance delivery, although one has been proposed within Sports Science [23].

Our study has highlighted that the workforce model and community setting was feasible and acceptable to the staff involved. A strength of our study is that our sampling strategy successfully recruited participants with a range of ages and experience. Qualitative research is an interpretive methodology that does not aim to be statistically representative of the whole: it aims to distil ideas from the essence of collected data. Thus, the rigor of our qualitative study hinged on our collaborative approach to analysis and the experience of the qualitative researcher. Our study has distilled ideas that would be useful in developing clinical practice for frail older adults undergoing joint replacement and for clinical education. Limitations are the reliance on semi-structured interviews to capture the perspectives of the treating staff alone, rather than a more ethnographic approach combining direct observation with interviews.

In conclusion our study has highlighted that the workforce model and community setting was feasible and acceptable to the staff involved. It has however highlighted some issues that raise questions for both clinical educators and researchers about the integration of research and practice-based experience and how professional craft knowledge that is core to professional practice can be taught. These related to two key areas of professionalism: first, the need to adopt a less paternalistic didactic approach when delivering care in a patient’s home compared to in the clinic environment utilising different skills and effective communication strategies. For clinical educators, the challenge is how to equip students with these skills and how to impact professional craft knowledge.

## Supplementary Information

**Additional file 1:** **Supplementary file.** SRQR Equator standards for reporting qualitative research [3].

**Additional file 2.** CORKA interview outline questions.

## Data Availability

The datasets generated and/or analysed during the current study are not publicly available due to fact that consent was not taken from participants to share their interview transcripts for this purpose. Other data are available from the corresponding author on reasonable request.
