# Supplementary material for: The challenges and gains of delivering a home-exercise intervention: a qualitative study of physiotherapists and physiotherapy assistants
Source: BMC Musculoskelet Disord. 2022 Sep 3;23:839. doi: 10.1186/s12891-022-05790-z (PMC9440491; doi:10.1186/s12891-022-05790-z)
Supplement: Supplementary file 2 — Additional file 2. CORKA interview outline questions. [file 12891_2022_5790_MOESM2_ESM.docx]

Additional file 2: CORKA interview outline questions

1. Can you tell me a bit about your previous experience treating people with knee replacement?
2. Can you tell what you think are the good and bad things about the intervention and what you like / dislike about it?
3. Any examples where treating in the patients’ homes rather than the department has made a difference?
4. Did the treatment environment have any direct impact on the treatment you gave?
5. Did you have any fears or worries about delivering the treatment // it being delivered by physiotherapy assistants?
6. How confident were you in the way the treatment was delivered?
7. What skills do you think you need to treat people in their own homes?
8. When we are asking people to treat away from the hospital what should we be preparing staff for?
9. Any other observations about your involvement with CORKA?
